# Simulation of Smart Home Activity Datasets

**DOI:** 10.3390/s150614162

**Published:** 2015-06-16

**Authors:** Jonathan Synnott, Chris Nugent, Paul Jeffers

**Affiliations:** 1 School of Computing and Mathematics, University of Ulster, Jordanstown, County Antrim BT37 0QB, UK; E-Mail: cd.nugent@ulster.ac.uk; 2 There4you, 46 Ballynanny Road, Banbridge, County Down BT32 4LQ, UK; E-Mail: pj.there4you@gmail.com

**Keywords:** monitoring, simulation, smart environments, visualization

## Abstract

A globally ageing population is resulting in an increased prevalence of chronic conditions which affect older adults. Such conditions require long-term care and management to maximize quality of life, placing an increasing strain on healthcare resources. Intelligent environments such as smart homes facilitate long-term monitoring of activities in the home through the use of sensor technology. Access to sensor datasets is necessary for the development of novel activity monitoring and recognition approaches. Access to such datasets is limited due to issues such as sensor cost, availability and deployment time. The use of simulated environments and sensors may address these issues and facilitate the generation of comprehensive datasets. This paper provides a review of existing approaches for the generation of simulated smart home activity datasets, including model-based approaches and interactive approaches which implement virtual sensors, environments and avatars. The paper also provides recommendation for future work in intelligent environment simulation.

## 1. Introduction

There is currently a global trend of population ageing. In 2010, the median age within the global population was 29 years. This has now been predicted to increase to 36 years of age by 2050. In addition, the population of older adults who are aged 60 years or older is predicted to increase from 841 million people in 2013 to over 2 billion in 2050. The proportion of older adults within the global population has increased from 8% in 1950 to 12% in 2013 and is predicted to increase to 21% by 2050 [1]. This trend of population ageing is as a result of reductions in fertility combined with increases in life expectancy. Global statistics indicate that fertility levels have declined from 5.0 children per woman between 1950 and 1955, to 2.5 children per woman between 2010 and 2015 [1]. This decline in fertility is expected to continue and is predicted to reach 2.2 children per woman between 2045 and 2050. Life expectancy at birth in more developed regions has increased from 65 years in 1950 to 78 years between 2010 and 2015 and is predicted to reach 83 years by 2045 to 2050. In less developed regions, life expectancy at birth has increased from 42 in less developed regions to 68 years between 2010 and 2015, and is predicted to increase to 75 years by 2045–2050 [1].

The increase in life expectancy is a positive situation; however, there are a number of related effects that require consideration. Older adults are subject to higher rates of cognitive and physical impairment than the younger population [2]. These conditions require long term monitoring and support in order to maximize quality of life and minimize progression where possible. The increased prevalence of such conditions will place an increased strain on healthcare resources. Older adults are also more prone to higher levels of sedentary behavior (SB), which is associated with several chronic risk factors [3,4]. Two related priorities in research are therefore: To establish novel and effective methods for assessment of activity levels in the home, and to establish novel methods for the long term monitoring and management of chronic conditions with the purpose of alleviating the increased strain on healthcare resources. The use of sensor technology within intelligent environments (IEs) is one such approach which has the potential to facilitate these needs. IEs can provide objective data describing behavior and health status, facilitating the development of novel activity recognition, assisted living, or healthcare monitoring solutions. Researchers require access to sensor data in order to develop novel solutions, however, the access to such datasets is limited [5]. This will ultimately slow the development of novel solutions [6]. Therefore, simulation of IE sensor data is a valuable area of research.

Section 2 provides background to the research, describing the impact of the ageing population and chronic conditions, providing an overview of existing IEs and existing data repositories. The section also discusses the need for IE data simulation. Section 3 provides an overview of existing IE data simulation approaches, with a particular focus on model-based and interactive approaches. The section also identifies several areas that would benefit from contribution. Finally, Section 4 provides concluding remarks.

## 2. Background

Many chronic conditions have a typical age of onset within the older adult age range, including chronic obstructive pulmonary disease, dementia, Parkinson’s disease, chronic heart failure and stroke, to name a few. Dementia is a progressive condition that has a number of symptoms including memory loss, mood changes, communication problems and eventually results in problems with the completion of everyday tasks once the condition reaches the later stages [7]. Prevalence of dementia also increases with age [8]. It has been estimated to have affected 35.6 million people globally in 2010 and this number is predicted to increase to 65.7 million by 2030 and 115.4 million in 2050 [9]. In the UK, dementia currently costs the National Health Service, local authorities and families £23 billion per annum [10]. These chronic conditions have no cure and require long-term, personal care [2]. In addition to the increased strain being placed on healthcare resources, there are also a number of issues with existing clinical practice for the diagnosis and assessment of certain chronic illnesses. The continuous assessment of physical and cognitive function can facilitate the early identification of a decline in health status, providing the opportunity for earlier intervention that may address and alleviate health problems before they have a significant impact on quality of life [11]. It is, however, costly and impractical to provide a sufficient level of specialized care facilities with the current predicted shift in demographics [12].

In addition to the issues with facilitating long-term continual assessment of those already suffering from cognitive or physical decline, there is also a growing interest in the long-term assessment of physical activity levels in the healthy population. Participation in low levels of physical activity or high levels of sedentary behavior SB may result increased prevalence of chronic conditions. The World Health Organization has provided global recommendations on the physical activity level needed for the prevention of noncommunicable diseases, depression and cognitive decline. For those aged 65 and above, it is recommended that at least 150 min of moderate-intensity or 75 min of vigorous-intensity aerobic physical activity are performed [13]. The intensity level of an activity can be expressed as Metabolic Equivalent of Task (METs), which is defined as a ratio of metabolic rate during a physical activity to the metabolic rate of the average person seated at rest. Moderate-intensity physical activities have an energy expenditure of approximately 3–6 METs, and include activities such as housework and domestic chores, brisk walking and gardening. Vigorous-intensity physical activities have energy expenditure greater than 6 METs, and include activities such as running, hill walking, and fast swimming [14].

SB is defined as waking behavior with an energy expenditure less than or equal to 1.5 metabolic equivalents (METs) performed while in a sitting or reclining posture [4]. SBs include activities such as watching television, using a computer, reading, and sitting while socializing [3,4]. Studies have shown that older adults engage in increased amounts of SB. For example, a study involving 6329 participants found that the amount of daily SB time increased in age groups above the age of 40. The mean hours per day of sedentary activity was 7.55 for those aged 40–49, 7.87 for those aged 50–59, 8.41 for those aged 60–69 and 9.28 for those aged 70–85 [15]. The health benefits of engaging in the recommended amounts of moderate to vigorous physical activity (MVPA) are well documented in literature. However, it is possible to engage in the recommended level of MVPA while still engaging in high levels of sedentary activity [3,4]. Emerging evidence indicates that SB may have an independent role on health, and it may still be possible to experience the risk factors associated with SB while achieving MVPA goals. Such risk factors include chronic, noncommunicable diseases such as cardiovascular disease, diabetes and cancer [3,4]. As a result, it has been suggested that there may need to be an adjustment in the prescription of the optimal daily human activity patterns for health [16]. There has previously been a lack of direct monitoring of SB levels, and it has been suggested that the monitoring of SB levels should receive as much attention as the monitoring of MVPA levels [17]. SB monitoring has therefore become a new focus for research on physical activity and health [18]. It is understood that through the long term, frequent monitoring of SB levels, it is possible to identify inadequate levels and invoke change before negative effects occur. As a result, this may reduce the occurrence of associated risk factors, further reducing strain on healthcare resources.

Several methods have been used for the recording of SB levels. These can be categorized as self-reported measurements and device-based measurements. Self-reported measurements involve the use of questionnaires or behavior logs to capture past behavioral trends. These approaches suffer from a range of issues, for example, questionnaires may suffer from random and systematic reporting errors, and the use of behavioral logs may be limited due to participant burden, systematic reporting errors and administration costs [19]. Recent research has focused on the development of novel device-based approaches for the objective and unobtrusive capturing of SB levels. Such approaches are predominantly based on the use of environment sensors, body-worn accelerometers, or computer vision technology.

### 2.1. Intelligent Environments

IEs are seen as one solution to facilitate long-term at-home monitoring of activities and chronic conditions. These are environments that are capable of monitoring their own state and the state of their inhabitants with the purpose of improving the experience within that environment [20]. Data collection within these environments is facilitated through the use of a range of sensor technology, chosen for the specific monitoring purposes of the environment and the needs of its inhabitants. The sensors incorporated into these environments are versatile, ranging from basic presence sensors capable of providing Boolean values specifying the detection of movement within a room, to more invasive solutions capable of identifying specific inhabitants and their actions [21]. Examples of sensing technology include simple sensors such as passive infrared (PIR) sensors or floor sensors capable of detecting movement within an area, contact sensors capable of inferring object or door interactions and pressure sensors embedded in chairs or beds capable of identifying occupancy. Environmental sensors such as temperature, light, humidity or barometric pressure sensors may be used to infer changes in the ambient properties of the environment. Computer vision or audio-based systems may provide additional information describing inhabitant behavior. These environments may incorporate the use of wearable technologies such as accelerometer or gyroscopes to provide movement measures. Additionally, bespoke systems consisting of several technologies developed for the monitoring of specific activities may be incorporated into such environments [21,22,23]. IEs can be incorporated into a wide range of environment types to serve many different purposes. For example, such environments have been used within office environments for the visualization of space utilization [24] and the movement of employees [25]. They have also been used within airports [26] and banks [27] for the detection of security risks indicated by abnormal behavior.

IEs deployed within the home environment are of particular relevance to the long-term monitoring of the ageing population. These environments, called Smart Homes (SHs), involve the use of the aforementioned sensor technology within the home environment to monitor the health, wellbeing, activities and security of inhabitants over extended periods of time [28]. The home-based monitoring facilitated by SHs provides accurate, reliable data with the potential to improve the medical condition of inhabitants through early detection of symptoms and may improve clinical effectiveness through decreased numbers of hospital admissions and reduced average length of stay [29]. Assessment of the long-term behavioral patterns of SH inhabitants may facilitate the early prediction of future health changes [11,30,31] through analysis of individual activity performances and the overall pattern of activity performance over extended periods of time [21]. Ultimately, these early warnings of health deterioration could facilitate timely intervention, extend the period of time a person can remain at home and reduce hospital admission [31].

SHs also have the advantage of facilitating ageing at home, commonly referred to as ageing in place. Despite the independence of many older adults being an issue due to the prevalence of age-related health challenges [28], many older adults would prefer to remain living at home for as long as possible as an alternative to using institutionalized care [20]. Findley *et al.* [32] reported that for those with Parkinson’s disease requiring long-term care, full-time institutionalized care resulted in a 450% increase in direct annual costs compared to those living at home. SHs are developed to address the desires of older adults to remain living independently while minimizing healthcare costs and the strain on healthcare services [12,20,21,28,31,33]. Pollack [2] divides the type of services such environments can offer into 3 categories: assurance, compensation and assessment. Assurance systems are developed to ensure the safety and wellbeing of vulnerable inhabitants by detecting harmful events [34] and providing regular status reports to caregivers. These may involve simple contact switches that may generate an alert if an inhabitant attempts to leave the SH during the night time, or may involve additional more complex processing with a network of sensors. For example, to infer that no meals have been prepared for a certain period of time, indicating that there may be an issue requiring intervention. Such measures may be required due to the high level of older adults living alone. It is estimated that 19% of older women and 11% of older men live alone [1]. Compensation systems provide guidance to inhabitants to help assist with the completion of activities. Assessment systems provide metrics describing cognitive or physical functioning based on continual assessment of activity performance.

Such assessments of activity performance may be facilitated through the analysis of general activity or SB levels, or the completion of activities of daily living (ADLs) [21]. ADL analysis is viewed by medical professionals as being one of the most effective methods of detecting emerging medical conditions [35]. ADLs include the actions performed during daily living in order to provide self-care, such as feeding, bathing, dressing and grooming [21,36]. Data provided by assessment systems may be processed to generate simple statistics such as mean and standard deviation (SD) of activity metrics for comparison over time, or more complex methods using artificial intelligence methods for classification of activities [21].

There is a wide range of SH implementations based in locations around the world including North America, Asia, Australia, Europe and New Zealand [21,37]. Prominent implementations include the PlaceLab created by the House_n research group at the Massachusetts Institute of Technology. The environment is in the form of a smart apartment containing a living room, dining area, kitchen, small office, and a bedroom, and is capable of facilitating data collection for individual or multiple inhabitants over multiple weeks. The environment supports sensors such as contact, pressure, temperature, humidity and light sensors [38]. The GatorTech SH located in Gainesville, Florida, was created by the University of Florida’s Mobile and Pervasive Computing Laboratory and College of Public Health and Health Professions. This SH was developed as a programmable pervasive space, designed to facilitate expandability and support for future sensor types by using a service layer to define each sensor and actuator within the space, facilitating future additions for new sensor types. The environment represents a typical home environment, containing a dining area, bedroom, bathroom, kitchen, living area, and a garage [22]. The TigerPlace project has been developed by the University of Missouri-Columbia as an ageing in-place facility for older adults, offering long-term housing for elderly inhabitants as an alternative to nursing home residency. The sensor technology incorporated includes a set of wireless infrared proximity sensors used to detect motion and presence within particular rooms, and for the identification of activity performance. For example, motion sensors placed within cabinets and refrigerators detect kitchen activities, a motion sensor installed in the ceiling above a shower detects showering activity and motion sensors above doorways can detect movement in and out of rooms. Other sensors include pressure pads, bed sensors capable of detecting presence, respiration rate, pulse and movement in bed, in addition to a passive gait monitor [11].

Washington State University’s Centre for Advanced Studies in Adaptive Systems (CASAS) [39] developed a “smart home in a box”, designed as a small, lightweight and extendable solution which is capable of performing key capabilities “out of the box”. The physical components of the solution are able to fit in a small box, containing sensors pre-labeled with intended locations, and a small low-powered server computer which hosts the middleware, database and application components. The available sensors include infrared motion detectors, door, temperature, and light sensors. This solution facilitated deployment of a large number of smart home testbeds. In 2009, 32 smart home testbeds had been deployed.

The Philips HomeLab is located in Eindhoven, The Netherlands. It represents a house with a living room, kitchen, two bedrooms, a bathroom and also a study. It is equipped with 34 cameras throughout the environment in addition to an observation room. The purpose of the environment is to generate data for behavior analysis and the identification of precursors or consequences of behavioral events [40]. The Ubiquitous Home was constructed in the Keihanna Human Info-Communication Research Center in Japan’s National Institute of Information and Communications Technology [41]. It consists of a living room, kitchen and dining room, a study, bedroom and bathroom. It was developed to facilitate the performance of context-aware service experiments through the use of cameras, microphones and various sensor technologies. The sensor technologies include floor pressure sensors, Infra-red sensors, accelerometers and Radio-Frequency Identification readers.

The Smart Environments Research Group at Ulster University, Northern Ireland has created a smart lab consisting of several rooms including a sensor equipped kitchen, living room and meeting room. The sensors deployed within the environment include a range of technologies such as X10 and Tynetec PIR, contact, chair pressure and floor pressure sensors, in addition to novel thermal sensing and eye tracking technologies [42].

### 2.2. The Need for Simulated Sensor Data Generation

Access to sensor datasets generated within IEs is essential for the testing of new approaches in a wide range of areas that utilize sensor data. For example, activity recognition approaches rely on test data for the assessment of the performance of new algorithms [5] and models [43], data-driven learning approaches [44] and to facilitate the selection of appropriate classification mechanisms [45]. Datasets are currently available from several IE projects. WSU’s CASAS project provides over 24 public datasets describing annotated and unannotated ADL performance including interweaved ADLs, multi-resident ADL activities, and daily activities in a range of environments including an apartment, a two-story home and an office building. The activities recorded include sleeping, bed-toilet transition, eating, taking medication, cleaning and relaxing [46]. Several datasets have been produced by van Kasteren [47]. The most cited of which is a dataset describing 28 days of annotated sensor data, including motion detectors, reed switches, cameras, accelerometers and RFID readers. The recorded activities include toileting, showering, sleeping and preparing meals [48]. While these datasets provide are a useful resource, researchers are required to generate their own datasets in order to facilitate the testing of specific environments, sensors and activities with novel analysis approaches.

Acquisition of such datasets is subject to limitations for a number of reasons [5]. Physical IEs are expensive to implement due to the cost of the sensor technology and the construction of the physical environments [5,45,49,50,51]. Additionally, the construction of such environments is a time consuming process requiring considerable groundwork before commitment to the purchase of equipment for the environment construction [52]. Prior to construction, however, researchers may not be aware of the ideal sensor configuration to achieve acceptable performance without the prior testing of several combinations of sensors [6]. This requires considerable time and expense and may be impractical in real-world implementations where significant alterations to the environment may lead to distress or confusion, particularly to inhabitants suffering from conditions such as dementia [52]. These environments therefore lack flexibility [51,53] and may have limited scalability [51]. As a result of these costs and constraints, not all researchers have direct access to such environments and the datasets they produce.

The collection of data from IEs is a time consuming process [5,45,49] due to the nature of the monitored scenarios, which may require the collection of data over extended periods of time in order to capture events that demonstrate typical inhabitant behavior and changes in behavior over time. Optimal testing of new approaches ideally involves the collection of data from all scenarios under all circumstances. This may not be possible in physical IE implementations due to the difficulty in recruiting suitable participants to test all scenarios [5,49] and certain situations may be unethical to test on vulnerable patients [52]. Additionally, there are regulatory limitations that must be adhered to during testing on human subjects [5].

These issues with the collection of IE sensor data are detrimental to research progress and are slowing down advances in the development of new approaches [6]. Researchers are therefore looking at alternative methods of IE data generation. One popular area of current research investigates the creation of novel methods for the generation of synthetic sensor datasets through the use of simulated IEs, which have been said to have the potential of accelerating research in related areas [5]. These simulated environments have the potential to facilitate the generation of vast sensor datasets, even larger than those from physical IEs [5]. Such simulations for the generation of data allow researchers to quickly test and evaluate new algorithms accurately and cost effectively [5,49] and provide an increased level of control over the environment and the data produced. The physical layout of environments including walls, doors and objects within the environment can be modified to test a range of use case scenarios. The arrangement of sensors including their type, number and position can be adjusted in terms of application scenarios as often as required with no cost and with very little time and effort [6,45]. Researchers are given complete control over the environment and the generated datasets [49], experiments can be re-run many times with small adjustments to the environment, catering for the refinement and fine tuning of the environment and the models or algorithms under development [5]. The experiments can be restarted quickly and easily with minimal setup time and there is complete control over the consistency of the state of the environment which is ensured in terms of the initial starting state and control over any anomalous events such as sensor failure [54]. Simulations facilitate control over environments which is not possible in real life, for example the manipulation of time, facilitating the rapid generation of datasets representing months or years of data [54]. These simulations may also facilitate the use of sensor technology that is expensive, difficult to obtain, or which is entirely conceptual and is yet to exist [54], facilitating the investigation into solutions that are built to be compatible with future technology. Simulations may represent entirely conceptual environments, or they may represent environments that already exist, indicating the impact of adjustments to the environment and highlighting optimizations in sensor placement with no invasiveness or expense [52]. As such simulations exist entirely digitally, they promote collaboration and open problem solving to a wider research community [5]. Studies that rely on simulation during the design phase are ultimately more likely to include more robust and inclusive designs [5].

## 3. Approaches for Smart Home Simulation

This section discusses two key categories of existing research within the area of IE data simulation: Model-based approaches that facilitate the generation of data based on activity models, and interactive approaches that incorporate the use of virtual environments (VEs) and virtual sensors which respond to user interaction. The following sub-sections describe each of these in further detail.

### 3.1. Model-Based Approaches

Model-based approaches for data simulation for the generation of synthetic sensor data involve the specification of activity models that define the order of events, the probability of events occurring and the time taken for each event during the performance of specific activities. Bouchard *et al.* [55] provided an example of such an approach used within the SIMACT SH simulator. This tool provided a form-based interface for the specification of scripts that detail the series of steps involved in the performance of activities within a SH. Users could define the order of events, the time taken for each event and the objects involved in the event. Additionally, users could define actions associated with the completion of each step, such as the movement of objects from one position to another or the rotation or scale adjustment of an object. Such actions are visualized within a 3D environment created separately within SketchUp [56]. Scripts could be replayed in real-time or fast-forwarded and could be replayed with adjustments to event timings. As scripts are played, object interaction data is stored within a database; however, details relating to the format of output data are limited. Bouchard *et al.* plan to provide an open source database containing example recorded scenarios. Mendez-Vazquez *et al.* [49] demonstrated the use of Markov chains describing the order of events, combined with Poisson distribution to calculate a range of realistic activity times and probability distributions to calculate a range of sensor values to generate a simulated activity dataset. This simulated activity set contained a distribution of activities such as reading, sleeping, walking and sitting together with metrics including time and energy expenditure. Another example of a model-based approach was demonstrated by Helal *et al.* [5] in the PerSim simulator. PerSim was developed to facilitate the synthesis of data for the testing of activity recognition research. The simulator allowed users to define activities by specifying the sensors involved in each activity, the order of sensor activations, the maximum and minimum typical sensor values and activity duration. Based on these parameters a list of sensor data could be generated in the Sensory Dataset Description Language. This synthesized dataset could contain data describing the result of individual activity performances, or for an entire space, including sensors not fired directly by activity performances, such as temperature sensors.

Kormányos and Pataki [57] developed a simulator capable of modelling the activity of a single inhabitant within an IE. The approach facilitated the modelling of individual behavior profiles, such as typical sleep amount, and the change in current state such as thirst and tiredness. The approach was capable of outputting data from simulated motion sensors, RFIDs and water consumption. The change in current state, such as thirst, influenced the likelihood of activity occurrence, such as drinking.

Model-based approaches have the potential to generate extensive simulated datasets describing activity performances over extended periods of time. The quality and accuracy of the resulting datasets, however, relies heavily on the quality of the activity description model and associated parameters. The construction of accurate activity models requires access to real test data describing the performance of the modelled activities. Additionally, it may be difficult to accurately and intuitively adjust such models to represent subtle yet significant differences in activity performance. For example, the impact of a phone ringing in a living room area during completion of the “making a cup of tea” activity in the kitchen, the impact of adjustments to environment and sensor layout on the quality of data generated, or the differences in activity performance between various IE inhabitants.

### 3.2. Interactive Approaches

Interactive approaches offer an alternative solution to IE simulation. Such approaches have a focus on user interaction with simulation software in order to provide fine-grained control over the activities and the resulting datasets. These approaches consist of software which provides a platform for interaction with individual virtual sensors, or the use of interactive VEs combined with embedded avatars that have the potential to provide an intuitive and interactive environment simulation experience. Avatars are interactive objects that can move within VEs and passively or actively interact with the sensors contained within them, representing the behavior of real inhabitants within physical IEs. These approaches rely on the modelling of environments and individual sensors rather than the modelling of activities. Such models may be based on existing environments or sensor specifications, or may be based on conceptual environments and technology which is yet to exist. Activities can be performed in a natural manner by interaction with a virtual sensor or movement of the avatar within the VE and interaction with objects contained within the VE. This facilitates ad-hoc testing [58] through the recording of specific activity scenarios, such as interruption during activity performance, falls, or the impact of subtle changes in object and sensor placement on the data generated. For example, a PIR sensor located in the far corner of a hallway may only detect inhabitants when they enter or leave the kitchen or living room and may not detect inhabitant movement between rooms further down the hallway. Additionally, adjustments to the layout of the environment, such as the placement of a table within a room may result in an adjustment of inhabitant movement paths, resulting in a PIR sensor or pressure sensor detecting inhabitant movement less often.

#### 3.2.1. Interactive Approaches for Context Aware Applications

Several studies have investigated the use of interactive VEs for the testing of context aware approaches. These studies do not focus on the output of synthetic data, but instead focus on illustrating the response of objects in the VE based on context aware criteria. While these approaches do not facilitate the generation of simulated sensor data, they have been included in this literature review as they are used for the prototyping of solutions for use within IEs.

Lertlakkhanakul *et al.* [58] describe the use of a 3D VE that supports interaction by multiple users simultaneously for collaborative exploration of the environment. This approach facilitated the configuration of SH automation services depending on user interactions within the environment. For example, the automation of room lighting changes when the user lies down in a bed. Fu *et al.* [53] demonstrated an avatar-based approach to SH simulation for the testing of context aware applications. They provided details of a simulator which represented a VE through the use of a 2D floor plan layout, capable of visualizing the current state of physical sensors within an IE, or visualizing the current state of virtual sensors through the use of text boxes placed next to sensor icons within the VE. Simulation of movement within a VE was supported by using the mouse to drag an avatar throughout the VE, generating position data. Sensors within the VE responded according to a set of context rules defined by the user in XML. For example, if the x position of the avatar exceeds a defined threshold, switch the living room light on. These avatar movements could be recorded and replayed to test alternative context scenarios; however, the authors provide limited details regarding the support for the creation of VEs and the support for generation of virtual sensor data.

The YAMAMOTO toolkit [59] facilitated modelling of environments using a “2.5 dimensional” format in which each floor in multi-level environments are represented as flat 2D floor plans connected by slopes representing stairs. Users trace outlines of rooms and corridors over a floor plan backdrop image, specifying whether each edge is passable and what type of boundary (for example wall, wall with window, wall with door) the edge represents and this meta-data can be used to generate 3D representations of the environment. The approach was used to simulate an assistive environment by placing a virtual proximity sensor capable of responding to the location of a user controlled avatar within the VE. This facilitated the simulation and testing of a real system that could detect Bluetooth devices to identify the presence of individuals at a kiosk, automatically retrieving an individual’s profile and displaying personalized content. The proximity sensor’s detection range was specified by a sphere radius and this virtual sensor was capable of generating an event identical to that of the physical sensor upon avatar detection.

Armac and Retkowitz [51] describe the eHomeSimulator, which represents environments graphically using a 2D overhead floor plan view. This VE is created in a grid format using SketchUp [56], which is then imported into the simulator to define accessible or inaccessible areas and to add devices and avatars. Multiple user controlled avatars can be placed in a VE and can be moved individually, facilitating the testing of automation services based on sensor input. For example, a service can be created called “music follows user”, in which a person’s favorite music will play in any room an avatar moves into. This simulator facilitates the testing of complex scenarios such as the event in which another person moves into the same room after this user. This allows for the testing of the correct outcome in such scenarios.

Other related approaches include 3DSim [60], which facilitates the testing of smart devices, CASS [61], which facilitates the testing of home automation rules, and TATUS [62], which facilitates avatar interaction through XML commands. Additionally, UbiREAL [63] facilitates the testing of ubiquitous devices within a range of contexts and UbiWise [64] facilitates the testing of embedded software for ubiquitous devices.

These studies have provided advances within IE research by facilitating rapid, low-cost testing of context aware approaches for environment automation. The studies facilitate the performance of simulated actions within VEs, allowing the user to compare VE automation behavior with the desired behavior.

#### 3.2.2. Interactive Approaches for Simulated Dataset Generation

Several studies have investigated the use of interactive approaches to facilitate the generation of realistic simulated IE sensor datasets.

Buchmayr *et al.* [45] introduced a simulator for the generation and visualization of sensor data within IEs. This simulator displayed a VE using a 2D floor plan layout and facilitated user interactions with virtual sensors through mouse clicks within the floor plan which generated sensor data output to a log file. The simulator supported the use of simple sensors such as binary, contact and temperature sensors that fire a signal upon activation and complex sensors such as motion and pressure sensors that fire a signal periodically after activation. The simulator also supported the generation of random data to simulate sensor faults. The addition of sensors to a floor plan was supported through dragging and dropping within the 2D floor plan; however the creation of new sensor types required development of data models, parsers and filters for each sensor, reducing accessibility of this area of functionality for non-technical users. This study did not facilitate avatar interaction and there are limited details about the process of VE creation or data visualization.

Several VE-based studies relating to the synthesis of IE sensor data have also used the avatar-based approach. Poland *et al.* [52] developed the SH Simulator, a tool which used a 3D VE approach to the simulation of inhabitant movements within an environment with the aim of facilitating the identification of optimal sensor placement before investment in physical sensors or real environment alterations. This simulator facilitated movement of an avatar throughout a VE using the keyboard and mouse, facilitating the generation of motion sensor and pressure sensor data. Movements could be recorded and replayed in order to test alternative sensor deployments. In this approach, the creation of environments and objects was completed using separate 3D modelling software including 3DS Max and GtkRadiant. A demonstration of the implementation of a VE involved the re-creation of the kitchen and living room area of the University of Ulster’s smart lab environment [42]. Users were able to view the environment from a first person, third person, or overhead view. The approach facilitated the adjustment of sensor properties including sensing radius, sensing angle and sensor texture. A similar tool was introduced by McGlinn *et al.* [65], which facilitated the simulation of location sensors within a 3D VE. VEs could be created using a game map editing tool and users were able to configure sensors by specifying accuracy, fire rate, delay and location using the SimConfig tool. Movements of avatars within the environment resulted in the generation of simulated data once an avatar’s position fell within a sensor’s detection range. This simulated data could be generic context or could be modelled to represent that of a real sensor type. The SimConViz tool provided a visualization of the VE and all avatars within it and provided feedback in relation to how the VE perceived the avatar movements based on the adjustment of sensor accuracy.

Krzyska [54] developed a smart house simulation tool that facilitated the creation of VEs using a 2D overhead plan layout presented using a simple color-coded line approach. Sensors and avatars were displayed as colored dots within the environment. The tool facilitated the placement of motion sensors with adjustable sensing radius. An avatar could be moved within the environment using mouse clicks, which generated sensor events within a log file if the avatar moved within a movement sensor’s detection radius. Scenarios involving multiple movements could also be recorded and replayed. Motion sensor position and sensing radius could be configured using a Form-based approach, however the tool provided no UI support for the creation of additional sensor types and sensor event logging adjustment required knowledge of the Log4J Java logging library.

Ariani *et al.* [66] developed an IE simulator which facilitates the creation of a floor plan and the specification of a resident profile for movement speed and height. An event scheduler facilitates the grouping of events in a scenario. Users can specify a start time, and the end time is automatically calculated. Event waypoints for movement within the environment can be specified, and a pathfinding algorithm is used to calculate valid movement. The simulation of events is able to produce PIR and pressure mat sensor data.

Synnott *et al.* [67] developed IE Sim, an intuitive, interactive approach to IE data simulation. This approach facilitated the creation of 2D overhead plans of VEs, allowing users to customize existing sensor types and create their own. The approach also facilitated the recording of complex ADL performance through passive and active avatar interaction with multiple objects and sensors within the environment. In a survey of 21 experts within the field of Ambient Intelligence, 90.48% of participants indicated that the software would be of use to them in their research [68]. IE Sim was then extended to facilitate the modelling of PIR event levels through probabilistic sampling. This provided some of the advantages of a model-based approach combined with an interactive approach by facilitating increased realism of generated PIR data, and facilitated a combination of manual avatar navigation with time-lapse functionality to generate datasets spanning extended periods of time [69]. Synnott *et al.* [70] later developed a thermal sensor simulator for the generation of low resolution thermal datasets. The simulator was designed to simulate a 16 × 16 thermal array sensor placed within the ceiling of an environment. Users were able to move multiple heat sources simultaneously throughout an environment through the use of a touch screen tablet. The software was developed in Unity3D [71], and was used for the initial development of an object tracking algorithm in MATLAB prior to the real sensor becoming available.

### 3.3. Existing Challenges and Opportunities for Contribution to Knowledge

Existing research in the area of virtual sensors and VEs has provided beneficial mechanisms for smart home data simulation. Nevertheless, there are several key challenges and opportunities for contribution to knowledge within the area. Many existing studies into VE-based interactive approaches focus on the testing of context aware applications [51,53,58,59,60,61,62,63,64], through the simulation of an environment and its reaction to user behavior rather than the generation of synthetic datasets. Significantly less VE-based interactive approaches focus on the synthesis of IE datasets [45,52,54,65]. Two existing approaches [52,65] have incorporated 3D VE approaches; however, such 3D VEs have been shown to be time consuming to construct [72]. The customizability and flexibility of existing approaches is limited. Some do not support the addition of new sensor types [52,54], or require programmatic implementation of new sensors [45].

The main focus of existing interactive approaches has been the simulation of activities completed by a single avatar, representing single occupancy within an environment. It is well known that the issue of multiple occupancy is a difficulty when developing novel activity recognition and inhabitant tracking approaches. In a survey of 21 experts within the field of Ambient Intelligence, 85.71% indicated in response to a questionnaire that the creation of an interactive approach that facilitates rapid and intuitive generation of multiple occupancy datasets would be of particular benefit to their research [68]. There is therefore an opportunity for novel interactive solutions which facilitate multiple occupancy data generation to provide a real benefit to the research community. The availability of mobile phones, tablets and laptops with touch screens capable of detecting multiple simultaneous touches provide an intuitive interaction mechanism for use with such approaches.

## 4. Conclusions

Access to sensor datasets is required for the testing of new data analysis approaches and the development of data driven activity recognition algorithms [5,43,45]. Availability of such datasets is, however, limited due to the high costs, constraints and limitations associated with the construction and usage of physical IEs [5,6,45,49,50,51,52,53]. The use of data simulation approaches has the potential to address these constraints, providing researchers with rich datasets for the testing of novel data analysis approaches, particularly at the early stages of development [5,6,45,49,52,54]. Existing IE simulation research has involved a range of approaches. Model-based approaches require the use of activity models and are capable of generating vast datasets representing extended periods of time. The accuracy of the data generated by these approaches depends on the accuracy of the underlying activity model, which may require existing datasets to derive and may not support ad-hoc testing. Interactive approaches offer the ability to record datasets with complete ad-hoc control over environment and sensor simulation, and may facilitate the assessment of subtle changes to environments or sensors, such as layout and sensor placement. A key area for future research into interactive simulation approaches is the development of techniques to facilitate the recording of detailed multiple occupancy datasets.
